# Effects of type and level of training on variation in physician knowledge in the use and acquisition of blood cultures: a cross sectional survey

**DOI:** 10.1186/1471-2334-5-71

**Published:** 2005-09-15

**Authors:** Jorge P Parada, David N Schwartz, Gordon D Schiff, Kevin B Weiss

**Affiliations:** 1 Midwest Center for Health Services and Policy Research, Hines VA Hospital, Hines, IL, USA; 2 Department of Medicine-Loyola University Medical Center, and the Stritch School of Medicine-Loyola University Chicago, Maywood, IL, USA; 3 Department of Medicine John Stroger Hospital of Cook County and Rush Medical College, Chicago, IL, USA; 4 Center for Healthcare Studies, and the Division of the General Medicine Feinberg School of Medicine, Northwestern University, Chicago, IL, USA

## Abstract

**Background:**

Blood culture (BCX) use is often sub-optimal, and is a user-dependent diagnostic test. Little is known about physician training and BCX-related knowledge. We sought to assess variations in caregiver BCX-related knowledge, and their relation to medical training.

**Methods:**

We developed and piloted a self-administered BCX-related knowledge survey instrument. Expert opinion, literature review, focus groups, and mini-pilots reduced > 100 questions in multiple formats to a final questionnaire with 15 scored content items and 4 covariate identifiers. This questionnaire was used in a cross-sectional survey of physicians, fellows, residents and medical students at a large urban public teaching hospital. The responses were stratified by years/level of training, type of specialty training, self-reported practical and theoretical BCX-related instruction. Summary scores were derived from participant responses compared to a 95% consensus opinion of infectious diseases specialists that matched an evidence based reference standard.

**Results:**

There were 291 respondents (Attendings = 72, Post-Graduate Year (PGY) = 3 = 84, PGY2 = 42, PGY1 = 41, medical students = 52). Mean scores differed by training level (Attending = 85.0, PGY3 = 81.1, PGY2 = 78.4, PGY1 = 75.4, students = 67.7) [p ≤ 0.001], and training type (Infectious Diseases = 96.1, Medicine = 81.7, Emergency Medicine = 79.6, Surgery = 78.5, Family Practice = 76.5, Obstetrics-Gynecology = 74.4, Pediatrics = 74.0) [p ≤ 0.001]. Higher summary scores were associated with self-reported theoretical [p ≤ 0.001] and practical [p = 0.001] BCX-related training. Linear regression showed level and type of training accounted for most of the score variation.

**Conclusion:**

Higher mean scores were associated with advancing level of training and greater subject-related training. Notably, house staff and medical students, who are most likely to order and/or obtain BCXs, lack key BCX-related knowledge. Targeted education may improve utilization of this important diagnostic tool.

## Background

"Of all the microbiologic procedures performed in the laboratory, few are as important as the prompt recovery of microorganisms from the blood."[1] – J.A. Washington, editor of *Infectious Disease Clinics*.

Per year, more than 200,000–250,000 blood stream infections occur in the United States, [2-5] and are the 10^th ^leading cause of death in the United States. [6] Bloodstream infection is associated with crude mortality rates as high as 50% in certain populations. [6-9] Bacteria enter the bloodstream indirectly via the lymphatic system with extravascular infections and directly with intravascular infections, and may present as transient (as with procedures or manipulation of infected tissues or mucosal surfaces or as with meningitis, osteomyelitis or pneumonia), intermittent (as with an undrained abscess) or continuous (as with endocardidtis and endovascular infections) bacteremia. Thus, the yield of blood cultures are related to the underlying infectious process and may be of limited utility at times. However, clinical management of infectious diseases depends on the accurate identification of the causal microorganism and its antimicrobial susceptibility, unusual organisms may be identified that may not be adequately treated by routine empirical coverage. [10] Because blood cultures (BCXs) represent the "gold standard" for the diagnosis of blood stream infections, their timely and appropriate utilization play a pivotal role in antimicrobial therapy. [11]

Blood cultures are a highly user-dependent diagnostic test. Optimal BCX yield – highest sensitivity and highest specificity – critically depends not only on the nature of underlying infectious process, but also on technique and timing of specimen acquisition. Proper aseptic technique has been shown to decrease the rate of contaminants. [12,13] The timing of BCX acquisition in relation to rigors/fevers and antibiotic administration also impacts on BCX yield. [13] The volume of blood, number and type of BCX bottles collected, likewise impact on sensitivity/specificity. [1,12-14]

Blood culture use is often sub-optimal. Standards published by the American Society of Microbiology indicate that the rate of contaminant BCXs should not exceed 3%. [15] Nevertheless, many teaching hospitals have rates that exceed 6%. [15,16] It has been estimated that each contaminant BCX adds $4,500 in additional cost (due to additional diagnostic testing, increased length of stay, unnecessary medication use and associated adverse events). [15] In addition, failures to obtain BCXs represent failed opportunities for guiding antimicrobial therapy. For example, 43% of empiric vancomycin courses in our institution lacked appropriate cultures, impeding efforts to streamline subsequent antimicrobial therapy. [17] Rapid identification and susceptibility testing of bacterial BCX isolates is also associated with more timely and cost-effective antibiotic therapy in hospitalized patients. [18]

Duration of medical training has been shown to impact resource utilization and diagnostic test use. [19] In teaching institutions like ours, inexperienced house officers and medical students are responsible for most BCX ordering and acquisition. The purpose of this study was to assess the associations between the level or type of physician training and of BCX-related knowledge among a wide range of physicians and physicians in training. We also sought to determine whether self-reported BCX-related training is a reliable indicator of measurable BCX-related knowledge.

## Methods

This is a cross sectional study using a self-administered survey instrument.

To develop a self-administered survey instrument to assess BCX-related knowledge and training, we began with a preliminary questionnaire containing > 100 evidence-based questions in multiple formats. Focus groups and mini-pilots were used to ensure face and content validity, and to guide an informal process of item reduction.

The prototype survey instrument used 32 knowledge-related multiple-choice and Likert scale questions. The BCX-knowledge questions covered various domains related to BCX use, including clinical indications for obtaining blood cultures, blood culture acquisition procedure, aseptic technique, optimal volume of blood for culture, number and timing of blood cultures, clinical indicators of BCX contamination, effect of antibiotic use on BCX utility, and usefulness of routine anaerobic cultures. All questions involved qualitative rather than quantitative judgments, thereby preventing bias in responses of caregivers for different patient populations (e.g., internists versus pediatricians).

We included four additional items to identify covariates of BCX-related knowledge. These included level of training, type of specialty training, and self-reported theoretical and practical BCX-related instruction. Sub-categories within each covariate group are delineated in Table 1.

**Table 1:** Selected Characteristics of Physicians.

Category of Training		Number (Percent)
Level of Training*	Attending	22 (9.2)
	≥ PGY3	115 (48.1)
	PGY2	19 (7.9)
	PGY1	27 (11.3)
	Student	17 (7.1)
Specialty/Type of Training^†^	Infectious Diseases	19 (7.9)
	Medicine	20 (8.4)
	Emergency Medicine	72 (24.7)
	Surgery	84 (28.9)
	Family Practice	42 (14.4)
	Obstetrics-Gynecology	41 (14.1)
	Pediatrics	52 (17.9)
Self-Reported Theoretical	Some	130 (44.7)
BCX-Related Instruction^†^	Little	96 (33.0)
	None	65 (22.3)
Self-Reported Practical	Some	156 (53.6)
BCX-Related Instruction^†^	Little	73 (25.1)
	None	62 (21.3)

* Total number = 291, students included

† Total number = 239, students excluded from total

We recruited infectious diseases specialists from a public teaching hospital, a private university medical center, and Chicago-area infectious disease specialists in private practice to guide item reduction and to establish a reference standard. This convenience sample of 23 infectious diseases physicians was recruited at a local infectious diseases conference, and their responses were used to establish an *expert opinion reference standard*. This reference standard was meant to establish a clinically useful approximation of optimal BCX-related knowledge and was established by tabulating the question-by-question infectious diseases specialists' responses of greatest frequency. To overcome issues related to any discordance in expert opinion, only responses with high concordance (95%) responses were included to establish the final *consensus opinion reference standard*. Thus, of the 32 BCX-knowledge items, 15 (47%) demonstrated a 95% consensus opinion by infectious diseases specialists and were retained in the final questionnaire [see Additional file 1]. In an item-by-item analysis of the 15 items, agreement with the reference standard was scored as 1+ (plus one) and disagreement as 1- (minus one). We summed these items to provide an overall *summary score *for each subject that was converted to a 0–100 scale.

We defined an *optimal knowledge standard *as responses matching evidence-based responses derived from our previous literature review. Of note, we found that both the expert opinion and consensus opinion reference standards matched the independently determined evidence-based optimal knowledge standard for all 15 items in the final survey instrument.

We administered the survey to a convenience sample of attending physicians, house officers and medical students of a large urban public teaching hospital. Most questionnaires were administered at conferences of the target services; specialties that use BCX infrequently (e.g. radiology, anesthesiology, psychiatry, dermatology, etc.) were not offered survey participation. Survey participation was voluntary and anonymous and the number of persons who did not complete or return the study is unknown. Respondents were not permitted to look up answers, and on average the survey questionnaire took < 5 minutes to complete. All responders were classified by level of training (medical student, interns, junior residents, senior residents and fellows, and attending staff), as well as type of specialty training (medicine, emergency medicine, surgery, obstetrics-gynecology, pediatrics, family practice and infectious diseases). Of note, interns, junior and senior residents, as well as fellows were classified according to their post graduate year (PGY) of training, as PGY1, PGY2, PGY3, or PGY ≥ 4.

Data were entered into a computerized database and analyzed using SPSS^® ^version 11.1 for Windows (SPPS Inc., Chicago, IL). Univariate analysis was conducted for each covariate. First we examined the mean item scores for each of the 15 scored items in the survey. Then, paired ANOVAs were used to examine the effect of level of training, type of specialty training, and self-reported theoretical and practical BCX-related instruction on the mean summary scores. Linear regression modeling was used to analyze the independent effects of these covariates on summary scores. Post Hoc testing included Bonferoni adjustment for multiple comparisons.

Approval for this study was granted by the Human Subjects subcommittee of the Institutional Review Boards of the study hospital and affiliate university. Participant confidentiality was maintained throughout the study. All members of the research team report no financial conflicts of interest with study participation.

## Results

Two hundred and ninety respondents met inclusion criteria and appropriately completed the survey. Table 1 provides a breakdown of responder characteristics by level of training, type of specialty training, and self-reported BCX instruction.

Level of training was found to be strongly and positively associated with BCX-related knowledge, with a difference of more than 17 points in the mean scores of attending physicians and medical students (85 versus 67.7 points, respectively; p < 0.001), and intermediate mean scores for resident physicians that improved with each additional year of training (Table 2). Infectious diseases physicians' scores of BCX-related knowledge were far higher than those of physicians from other specialties (p < 0.001), among which internal medicine physicians scored the highest (Table 2). Higher levels of self-reported theoretical and practical BCX-related instruction were similarly correlated with higher scores for BCX-related knowledge (Table 2).

**Table 2:** Physician BCX Knowledge Summary Scores by Level of Training, Self-Reported Theoretical or Practical Blood Culture Related Training, and Type of Specialty Training.

Category	Mean Score*	Score STD^†^	Range	P-value
Level of Training				
Attending^‡^	85.00	12.00	53.33–100	—
≥ PGY3	81.10	11.20	40.00–100	0.04
PGY2	78.40	14.10	20.00–100	0.01
PGY1	75.40	10.83	53.33–100	≤ 0.001
Student	67.70	15.63	26.67–100	≤ 0.001
Specialty/Type of Training				
ID^‡^	96.07	6.07	80.00–100	— (≤ 0.001)^§^
Medicine	81.73	12.37	20.00–100	≤ 0.001 (—)
Emergency Med	79.63	9.30	66.67–100	≤ 0.001 (0.483)
Surgery	78.53	13.50	40.00–100	≤ 0.001 (0.233)
Family Practice	76.47	9.47	53.33–100	≤ 0.001 (0.095)
Ob-Gyn	74.40	9.50	60.00–100	≤ 0.001 (0.015)
Pediatrics	74.00	8.33	60.00–100	≤ 0.001 (0.008)
Students	67.70	15.63	26.67–100	≤ 0.001 (≤ 0.001)
Self-Reported Theoretical Instruction				
Some^‡^	81.38	13.53	20.00–100	—
Little	78.26	12.77	53.33–100	0.081
None	73.33	14.95	26.67–100	≤ 0.001
Self-Reported Practical Instruction				
Some^‡^	80.90	13.14	20.00–100	—
Little	78.72	13.32	53.33–100	0.241
None	72.13	14.78	26.67–100	0.001

* Scale of Mean Summary Scores = 0–100

† STD = Standard Deviation

‡ Comparison group for the paired ANOVA (see P-values), ID = Infectious diseases

§ (P-value using Medicine as the comparison group)

Level of training and type of specialty training accounted for most of the variability in scores when all covariates are entered into general linear regression modeling (Table 3). After controlling for level of training and type of specialty training, self-reported theoretical and practical BCX-related instruction largely ceased to account for statistically significant changes in scores. In contrast, after controlling for level of training, statistically significant differences in scores persisted for all services as compared to infectious diseases (p < 0.001, Table 2, infectious diseases specialists as the comparison group). General linear regression modeling revealed that the categories infectious diseases, obstetrics-gynecology, pediatrics, PGY1 and student accounted for most of the variability in scores (Table 3).

**Table 3:** Multivariate Analysis of Physician BCX Knowledge Summary Scores in Model Including Level of Training, Type of Specialty Training, and Self-Reported Theoretical and Practical Blood Culture Related Training.

Category	P-value	95% Confidence Interval of Difference in Scores with Reference Group
Level of Training		
Attending*	—	—
> PGY3	0.181	-2.385, 0.452
PGY3	0.808	-1.641, 1.279
PGY2	0.081	-2.805, 0.162
PGY1	0.016	-3.379, -0.350
Student	< 0.001	-6.271, -3.224
Specialty/Type of Training		
Infectious Diseases	< 0.001	1.691, 5.305
Medicine*	—	—
Emergency Medicine	0.255	-2.867, 0.762
Surgery	0.180	-2.609, 0.492
Family Practice	0.099	-3.440, 0.299
Ob-Gyn	0.005	-4.405, -0.780
Pediatrics	0.015	-3.991, -0.439
Self-Reported BCX-related Training		
Practical Training	0.347	-0.327, 0.925
Theoretical Training	0.529	-0.430, 0.835

* Excluded due to co-linearity

**Figure 1:**
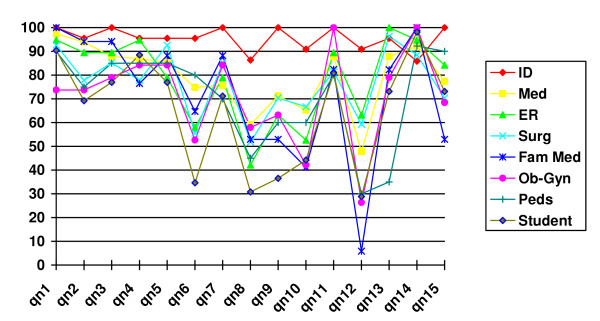
Item-by-Item Performance (Mean Score) by Type of Specialty Training/Service (Note: Seven questions (qn) – 6, 8, 9, 10, 12, 13 and 15 – accounted for the greatest variability in responses).

Examined item-by-item, all 15 questions were found to both match an evidence-based optimal knowledge standard and covered all previously identified relevant domains of BCX-related knowledge. Figure 1 details the item-by-item variability in services' responses to the 15 questions. Many items demonstrated similar high scores among respondents. Seven items had marked variability in responses; with the greatest number of incorrect answers they contributed the most strongly to the variability in overall scores.

## Discussion

Our data suggests that substantial variation exists among physicians in BCX-related knowledge and that this knowledge is significantly associated with the level and type of training. Apart from infectious diseases physicians, we identified important deficiencies in BCX-related knowledge among all specialties and at all levels of training. Of particular concern was the finding that scores of BCX-related knowledge were lowest among interns and medical students who are most often responsible for ordering and obtaining blood specimens for testing. In addition, approximately one half of respondents reported little or no practical or theoretical instruction in BCX utilization (Table 1). It is notable that fellows and attendings with infectious diseases specialty training had far higher mean summary scores than all other providers (p < 0.001 for all comparisons). It appears that there may be a specific body of knowledge that is commonly acquired during even early stages of infectious diseases training. Together, these results suggest that educational interventions to increase BCX-related knowledge may be an effective strategy for improving utilization of this important diagnostic test.

These data also suggests that self-reported instruction in practical and theoretical BCX-related knowledge correlate with overall BCX-related knowledge. This effect is largely explained by other factors like level of training and type of specialty training. However, at the lower training levels (student and intern), perceived BCX instruction remain a statistically significant predictor of score and may be a reliable independent measure of overall BCX knowledge. This may have implications for medical education as this suggests a simple direct query about perceived BCX instruction may help identify trainees who may benefit from additional education in BCX use.

In addition, these data show that physicians at higher levels of training (attendings) have greater BCX-related knowledge than physicians at lower levels of training and medical students. Admittedly, this conclusion seems intuitively logical, yet to our knowledge this is the first study that specifically investigated this relationship in this area. Furthermore, it might not have been surprising to find a very different relationship; one where those most directly involved with BCX acquisition (interns and residents) scored higher than students (lacking experience) or attendings (distanced from direct experience). These findings highlight an interesting paradox: the providers most directly involved ordering and obtaining BCXs (house staff and medical students) are the least knowledgeable about their indications and use. There appears to be a clear mismatch of these providers and those who are most knowledgeable about BCXs (attendings). We hypothesize this may contribute to sub-optimal BCX use, and wonder if better education early in training may improving knowledge and perception of BCX utility. As seven of the fifteen questions were found to have much greater variability in their responses (Figure 1), it is possible that an even more user friendly, reduced item, questionnaire may be deployable. These questions focused on the timing of specimen acquisition with regard to symptoms, number of cultures, and volume of blood for optimal blood culture yield, as well querying the respondents' understanding of the underlying infectious processes' likelihood of producing high grade bacteremia (i.e., endovascular infections versus pneumonia or cellulits) and when most blood cultures convert from negative to positive.

We believe these findings show that an area-specific (BCX-related) knowledge survey can be used to identify and target providers who might most benefit from educational interventions. In addition, measures of BCX-related knowledge could be used to assess if BCX-related knowledge predicts appropriateness of BCX acquisition behavior. Most importantly, this model might be extended to other areas of medical training.

A number of study limitations are worth noting. This is a single centre study, so generalizability of these findings remains unproven. In our approach to data analysis, some infectious diseases attending responders served for both to establish the reference standard and were subsequently included in the database. Understandably, if scores were derived from a comparison to this reference standard, the high scores of the infectious diseases service can be construed to be a self-fulfilling prophecy. However, a comparison of reference standard responses (be it expert opinion or consensus opinion standards) to evidence-based optimal knowledge showed all reference standard responses matched evidence-based responses. Therefore, the reference standard does not introduce a bias away from evidence-based optimal responses. However, we believe it does serve to establish a clinically based practical scoring system. An examination of infectious diseases fellows' responses supports this view. Infectious diseases fellows' responses were not used to establish the reference standard. Yet, the infectious diseases fellows' mean summary score was 90.5. This is higher than any other category in any service or any other level of training (except infectious diseases attendings). We believe this reflects that certain medical training does lead to increased BCX-related knowledge. This increased knowledge is reflected in higher mean summary scores and a closer approximation of optimal evidence-based knowledge. Finally, the survey was administered to a convenience sample primarily captured at conferences of target services, and unfortunately the participant response rates are unknown. We recognize the potential bias that may be associated with selective sampling of responders, and while we are encouraged by the strength of our findings, expanded meticulous surveying at multiple centers are needed to confirm these findings. We also recognize differential motivation of respondents may influence our findings. Infectious disease trained respondents perhaps felt more compelled to perform on this survey than others. However, most physicians are competitive individuals and we expect that those disinclined to perform well would also be disinclined to complete the survey. We know all infectious disease participants complete and return the survey while not all participants from other groups did. Thus, it is more likely that a selection bias towards a subpopulation of more motivated and higher scoring participants would be amongst non-infectious disease trained respondents.

Additional work is recommended. It would be important to investigate the link between BCX-related knowledge and actual physician behavior with regard to appropriate BCX acquisition. In addition, of great importance in this age of significantly decreased physician phlebotomies would be to broaden the focus to include non-physician phlebotomy and nursing staff which more and more commonly obtain BCXs on physicians orders. Appropriate blood culture acquisition could improve the sensitivity and specificity of BCXs. Improved diagnostic outcomes could be achieved through increased indicated phlebotomy, reduced unnecessary phlebotomy, as well as improved aseptic technique and decreased number of contaminants. Potentially, this could contribute to reductions in empiric antibiotic use, cost of treatment, and unnecessary use of hospital resources (including syringes, culture bottles, laboratory staff and equipment) and shorten hospital length of stay.

Finally, it there is the opportunity to extend the concepts of this work to other areas of medical care. This approach can be applied to other medical domains, as well as non-physician health care providers, to help better understand the relationship of professional training and the use of diagnostic or therapeutic interventions; or to screen persons for targeted educational interventions.

## Conclusion

Our data suggests that, as expected, level of training and type of specialty training is related to BCX-related knowledge. However, it appears that knowledge is not similar across physician specialties. Specifically, average knowledge scores of providers from medical, surgical and emergency medicine services were higher than those of providers from pediatrics, obstetrics-gynecology, and family medicine. At the higher levels of training, where the type of specialty training exerts greater impact, self-reported BCX-related instruction does not act as an independent predictor of BCX-related knowledge. However, at the lower training levels (student and intern), perceived BCX instruction may be a reliable independent measure of overall BCX knowledge. As such, it may serve as a screen to identify trainees who might benefit from targeted educational interventions. Improvement in knowledge deficits around BCX use may provide opportunities for better use of this important diagnostic tool, and ultimately improved patient outcomes.

## Abbreviations

BCX – Blood Cultures

PGY – Post Graduate Year

## Competing interests

The author(s) declare that they have no competing interests.

## Authors' contributions

All authors were responsible for study conception and design. JPP, GDS, DNS undertook the literature review. JPP collected the data and was responsible for the data management and analysis. All authors contributed to the interpretation of the study findings. JPP wrote the first draft of the paper and all the authors contributed to further drafts and critical review of the manuscript.

## Pre-publication history

The pre-publication history for this paper can be accessed here:



## Supplementary Material

Additional File 1 Survey Instrument. The BCX-related Knowledge Survey Instrument (includes the 15 scored items, and the four co-variate identifiers). Click here for file
