# Supplementary material for: Effects of type and level of training on variation in physician knowledge in the use and acquisition of blood cultures: a cross sectional survey
Source: BMC Infect Dis. 2005 Sep 15;5:71. doi: 10.1186/1471-2334-5-71 (PMC1261264; doi:10.1186/1471-2334-5-71)
Supplement: Additional File 1 — Survey Instrument. The BCX-related Knowledge Survey Instrument (includes the 15 scored items, and the four co-variate identifiers). [file 1471-2334-5-71-S1.doc]

**Additional file 1.** The BCX-related Knowledge Survey Instrument (includes the 15 scored items, and the four co-variate identifiers).

**Blood Culture Survey**

We greatly appreciate your participation in our survey. On the following pages are a few questions exploring the beliefs and attitudes of housestaff, attendings and students with regard to the utility of BCXs. **This is not a test**. Please CIRCLE the response that most closely approaches your beliefs about BCXs. **The answers you provide will be kept strictly confidential, and will NOT be traced back to you as an individual**.

PLEASE NOTE: Unless otherwise specified, assume:

**“BCX”** = Blood Culture.

**“BCXs”**= 2 sets of blood cultures (4 bottles: 2 aerobic, 2 anaerobic)

**“set”** of blood cultures = 2 bottles: one aerobic and one anaerobic bottle.

**“positive”** blood culture = any (one or more) bottle from the set(s) grow bacteria

(1) Service: ( )Medicine ( )Surgery ( )Ob-Gyn ( )E.R. ( )Other (please specify): _________

(2) Level of training: ( )Medical Student ( )PGY 1 ( )PGY 2 ( )PGY 3 ( )≥ PGY3 ( )Attending

(3) How much formal training have you had on theories and factors that effect BCX utility or yields?

(a) Some (b) Little (c) None

(4) How much formal, supervised, training on BCX technique (shown/taught how to draw BCXs properly) have you had? (a) Some (b) Little (c) None

Strongly Do not Strongly

Disagree know Agree

(1) The BCXs yield is affected by the (recent) prior use of antibiotics. 1 2 3 4 5

(2) In the setting of suspecting bacteremia, the chances of obtaining at least one 1 2 3 4 5

positive BCX result improve if more sets of BCXs are sent.

(3) Obtaining two or more sets of BCXs help distinguish between 1 2 3 4 5

contamination and true positive BCXs.

(4) A BCX set should consist of one aerobic bottle and one anaerobic bottle. 1 2 3 4 5

(5) Usually, contamination is during blood collection. 1 2 3 4 5

(6) The BCX yield depends upon volume of the blood placed in each BCXs bottle. 1 2 3 4 5

(7) The BCXs yield depends upon the timing of blood collection with regard 1 2 3 4 5

to symptoms (e.g. chills, rigors, fever).

(8) The ideal time to collect blood for BCXs is during rigors. 1 2 3 4 5

(9) The % of positive BCXs in endocarditis is more than in cellulitis. 1 2 3 4 5

(10) The % of positive BCXs in pneumonia is more than in a catheter/line infection. 1 2 3 4 5

(11) BCXs are a very good diagnostic tool in the setting of suspected sepsis. 1 2 3 4 5

(12) What is the ideal practical number of blood culture sets that should be sent each time BCXs are obtained? (a) one set (b) two sets (c) three sets

(13) What is the ideal time to collect blood for blood culture?

(a) during chills (b) during rigors (c) during fever spike

(14) Of all positive blood cultures, what percentage are contaminated (not reflective of bacteremia)?

(a) 0-15% (b) 15-30% (c) 30-45% (d) >45%

(15) Of all positive blood cultures, when do most convert from negative to positive?

(a) 0-24hrs (b) 24-48hrs (c) 48-72hrs (d) >72hrs

**⇒ ⇒ ⇒** Thank you for participating in our survey.
